# TBX15/miR-152/KIF2C pathway regulates breast cancer doxorubicin resistance via promoting PKM2 ubiquitination

**DOI:** 10.1186/s12935-021-02235-w

**Published:** 2021-10-18

**Authors:** Cheng-Fei Jiang, Yun-Xia Xie, Ying-Chen Qian, Min Wang, Ling-Zhi Liu, Yong-Qian Shu, Xiao-Ming Bai, Bing-Hua Jiang

**Affiliations:** 1 Department of Pathology, Nanjing Medical University, 140 Hanzhong Road, Nanjing, 210029 China; 2 The Academy of Medical Sciences, Zhengzhou University, Zhengzhou, 450052 Henan China; 3 Department of Medical Oncology, Thomas Jefferson University, 1020 Locust Street, Philadelphia, PA 19107 USA; 4 Department of Oncology, The First Affiliated Hospital of Nanjing Medical University, 300 Guangzhou Road, Nanjing, China; 5 Department of Pathology, Anatomy and Cell Biology, Thomas Jefferson University, 1020 Locust Street, Philadelphia, PA 19107 USA

**Keywords:** TBX15, miR-152, KIF2C, PKM2, Doxorubicin resistance, Breast cancer

## Abstract

**Background:**

Chemoresistance is a critical risk problem for breast cancer treatment. However, mechanisms by which chemoresistance arises remains to be elucidated. The expression of T-box transcription factor 15 (TBX-15) was found downregulated in some cancer tissues. However, role and mechanism of TBX15 in breast cancer chemoresistance is unknown. Here we aimed to identify the effects and mechanisms of TBX15 in doxorubicin resistance in breast cancer.

**Methods:**

As measures of Drug sensitivity analysis, MTT and IC50 assays were used in DOX-resistant breast cancer cells. ECAR and OCR assays were used to analyze the glycolysis level, while Immunoblotting and Immunofluorescence assays were used to analyze the autophagy levels in vitro. By using online prediction software, luciferase reporter assays, co-Immunoprecipitation, Western blotting analysis and experimental animals models, we further elucidated the mechanisms.

**Results:**

We found TBX15 expression levels were decreased in Doxorubicin (DOX)-resistant breast cancer cells. Overexpression of TBX15 reversed the DOX resistance by inducing microRNA-152 (miR-152) expression. We found that KIF2C levels were highly expressed in DOX-resistant breast cancer tissues and cells, and KIF2C was a potential target of miR-152. TBX15 and miR-152 overexpression suppressed autophagy and glycolysis in breast cancer cells, while KIF2C overexpression reversed the process. Overexpression of KIF2C increased DOX resistance in cancer cells. Furthermore, KIF2C directly binds with PKM2 for inducing the DOX resistance. KIF2C can prevent the ubiquitination of PKM2 and increase its protein stability. In addition, we further identified that Domain-2 of KIF2C played a major role in the binding with PKM2 and preventing PKM2 ubiquitination, which enhanced DOX resistance by promoting autophagy and glycolysis.

**Conclusions:**

Our data identify a new mechanism by which TBX15 abolishes DOX chemoresistance in breast cancer, and suggest that TBX15/miR-152/KIF2C axis is a novel signaling pathway for mediating DOX resistance in breast cancer through regulating PKM2 ubiquitination and decreasing PKM2 stability. This finding suggests new therapeutic target and/or novel strategy development for cancer treatment to overcome drug resistance in the future.

**Supplementary Information:**

The online version contains supplementary material available at 10.1186/s12935-021-02235-w.

## Background

Breast cancer is one of the most common cancers in women [1–3]. Resection and chemotherapy are used to improve survival, yet many patients develop chemoresistance which often leads to the recurrence of the disease. Doxorubicin (DOX) is an anthracycline chemotherapy agent that is effective in treating a wide range of malignancies, including breast cancer [4]. However, DOX resistance and a dose-related cardiotoxicity leads to therapeutic failure in a subset of patients [4]. Thus, it is important to identify new mechanisms that circumvent DOX chemoresistance to improve breast cancer therapy and patient survival.

TBX15 belongs to the T-box family of genes, which encode a phylogenetically conserved family of transcription factors to regulate a variety of developmental processes [5–7]. Genome-wide association studies have shown that TBX15 gene correlated with differentiation and development in inflammation or neoplastic lesions [8–10]. TBX15 promoter methylation or lower TBX15 expression are found in ovarian cancer [11], pancreatic cancer [6, 7], and hepatocellular carcinoma [8]. However, role and mechanism of TBX15 in breast cancer is unknown. We found expression levels of TBX15 were downregulated in DOX-resistant breast cancer tissues, and were strongly correlated with miR-152 levels.

Our previous study showed that the downregulation of miR-152 was associated with tumor grade and metastasis in breast cancer patients, whereas overexpression of miR-152 inhibited cell proliferation and tumor angiogenesis [12].

Using bioinformatics methods, we found Kinesin family member 2 C (KIF2C) is a potential target of miR-152. KIF2C is a critical microtubule regulator which induces disassembly of microtubules in an ATP-dependent manner and which is important for chromosome segregation during anaphase [13]. KIF2C elevated levels were observed in some types of cancer [14]. KIF2C may promote cell growth, metastasis, and chemoresistance in solid tumors through modulating p53 or suppressing microtubule dynamics [15–17]. But the role of KIF2C in breast cancer is unknown.

Pyruvate kinase M2 (PKM2), is a glycolytic enzyme that promotes the Warburg effect (e.g., anaerobic glycolysis), and is strongly upregulated in many cancer tissues as it contributes to cell proliferation, migration and metastasis through enhancing glycolysis [18–22]. In addition, it manifests drug resistance by modulating the balance of metabolic energy [22, 23]; PKM2 dysregulation may contribute to DOX resistance in liver cancer by regulating gulcose metabolism [23]. Previously, we showed that PKM2 is involved in miR-152-mediated suppression of cell proliferation and angiogenesis in breast cancer [12]. However, it is unknown if PKM2 and KIF2C may interact to affect the cancer development.

In this study, we plan to determine the roles and mechanism of TBX15/miR-152/KIF2C pathway on DOX resistance, and whether PKM2 is involved; to test whether TBX15 downregulation is sufficient to induce DOX resistance in breast cancer; and to define role and mechanism of KIF2C and PKM2 in mediating DOX resistance.

## Methods

### Cell culture and reagents

Human embryonic kidney 293 T (ATCC, CRL-3216), Mammary epithelial cells MCF10A cells (ATCC, CRL-10317) and Human breast cancer cell line T47D (ATCC, HTB-133) were obtained from American Type Culture Collection (Manassas, VA, USA). Human breast cancer cell line MCF7/ADR were obtained from Cell Bank of Chinese Academy of Sciences (Shanghai, China). MCF7/ADR and HEK-293 T were cultured in Dulbecco’s modified Eagle’s medium (DMEM) supplemented with 10% fetal bovine serum, penicillin (100 U/ml), and streptomycin (100 ng/ml). MCF10A cells were maintained in DMEM/F12 supplemented with 0.02 µg/ml Epidermal Growth Factor (EGF), 5% Horse Serum, 0.1 µg/ml CholeraToxin, 0.5 µg/ml Hydrocortizone and 1 µg/ml Insulin. T47D cells were cultured in RPMI-1640. Doxorubicin (DOX)-resistant T47D cells (T47D/ADR) were was developed based on T47D/WT cells. Starting from 1/10 of the IC50, the DOX concentration in the medium was gradually increased after the cells were stably grown. All cell lines were maintained in a 37 °C incubator with 5% CO_2_. All medium, including DMEM (11995040), DMEM/F12 (11320033), RPMI-1640 (11875085), and antibiotics (10378016) were obtained from Invitrogen (Carlsbad, CA, USA). Fetal bovine serum (A3520502) and horse serum (16050122) were obtained from Gibco (Carlsbad, CA, USA). EGF (SRP3196), CholeraToxin (C8052), Hydrocortizone (H1425000), Insulin (I2643), and DOX (D1515) were obtained from Sigma-Aldrich (St. Louis, MO, USA). STR profiling and mycoplasma contamination were performed to keep the authenticity of cell line on regular basis.

### Animals

All animals (female nude mice) were treated in accordance with the guidelines of the Institutional Animal Care and Use Committee at Nanjing Medical University. The animals were fed food and water *ad libitum*, and housed at 22 °C with a 12-h light-dark cycle. The normal diet was from Xietong Biotechnology Co. Ltd (Jiangsu, China). All animal experiments were carried out in accordance with the National Institutes of Health guidelines for the care and use of Laboratory animals (NIH Publications No. 8023, revised 1978). All animal studies comply with the ARRIVE guidelines.

In the end, the nude mice were euthanized using CO_2_ overdose. Put the mice into the sealed box, open the valve of 40% CO_2_ cylinder, keep the flow rate 0.6–0.8 L/min for about 1 min. In case of death symptoms, continue perfusion for about 1 min, and close the pressure reducing valve and cylinder valve in turn. After closing the valve, let the mouse stand for 2 min and confirm that it has no breathing and no heartbeat before removing the animal body from the sealed box. All animal experiments were performed in line with the Animal Care and Laboratory Guidelines and approved by the Ethics Committee of Nanjing Medical University and Jefferson University.

### Plasmid, miRNA mimics and siRNA transfections

The pCMV-based plasmid encoding human KIF2C or TBX15 and pcDNA3-based plasmid encoding human PKM2 were obtained from the nonprofit plasmid repository, Addgene (Cambridge, MA USA). The miRNA mimics were obtained from ribobio Biotechnology Co., Ltd (Guangzhou, China) and siRNAs were from Dharmacon ON-TARGET plus SMART pools. KIF2C siRNAs contained: ON-TARGET plus SMART pool siRNA J-004955-6, J-004955-7, J-004955-8, and J-004955-9. TBX15 siRNAs contained: ON-TARGET plus SMART pool siRNA J-022116-17, J-022116-18, J-022116-19, and J-022116-20.

The cells (2 × 10^5^) were seeded and grown in 6-well culture plates for 24 h before transfection with the above plasmids, miRNA mimics, or siRNAs using Lipofectamine 3000 (Invitrogen, Carlsbad, CA, USA).

### Drug sensitivity analysis

Cells in 100 µl medium were seeded (1 × 10^4^ cells/well) into 96-well microtitre plates. After 24 h, DOX in graded concentrations was added to the wells. 5 mg/ml MTT was added into each well to detect cell viability. Cell viability was calculated by dividing the OD590 absorbance value. The half maximal inhibitory concentration (IC50) values were analyzed using GraphPad Prism 7 software.

### Apoptosis assays

After various treatments, the cells were digested and resuspended in PBS. Cells were stained with Annexin V and DAPI and apoptosis status was measured by flow-cytometry analysis.

### Total RNA isolation and quantitative real-time PCR analysis

Total RNA was isolated using the Trizol reagent, according to the manufacturer’s instructions. For each sample, 0.5 µg of RNA was reverse transcribed using the PrimeScript RT Reagent Kit (TAKARA Bio Inc, #RR037A, Shiga, Japan). Real-time PCR analysis was performed using the Power SYBR Green PCR master mix (Roche Diagnostics, #04913914001, Indianapolis, IN, USA). All treatments and conditions were performed in triplicate to calculate statistical significance.

### Western blotting

Cells were treated with pharmacological agents for various times. The cells were collected into lysis buffer. Equal amounts of total proteins (20 µg) were separated by SDS-PAGE and transferred onto a nitrocellulose membrane. The membranes were probed with the appropriate antibodies. The immunoreactivity was detected by ECL and analyzed using Image Lab software. The following were commercially obtained antibodies: the anti-PKM2 (#4053s), the anti-p62 (#8025), anti-LC3 (#12741) and anti-Flag (#8146s) antibodies were obtained from Cell Signaling Technology (Danvers, MA, USA); the anti-KIF2C antibody (#sc-81305) were obtained from Santa Cruz Biotechnology; the anti-β-actin antibody was obtained from Bioworld Technology (Atlanta, Georgia, 305, USA).

### Co-immunoprecipitation

Cells were lysed in NETN buffer (20 mM Tris, at pH 8.0, 100 mM NaCl, 1 mM EDTA and 0.5% NP-40). For all samples, 1000 µg lysate was incubated with Protein G Sepharose beads and then followed by the indicated antibodies according the manufacturer’s instruction. Immunoprecipitates were resolved by immunoblotting with indicated antibodies.

### Lentiviral infection

The sequences encoding miR-152 and TBX15 were synthesized and cloned into the lentivirus vectors (Addgene, Palo Alto, CA, USA). To produce the virus, the plasmids were co-transfected into HEK293T cells, according to manufacturer’s instructions. To obtain a stable miR-152- or TXB15-overexpressing cell line, lentivirus-containing supernatant was harvested and used to infect MCF7/ADR cells.

### Tumor xenograft models

Four-week-old female nude mice were injected into both sides of posterior flanks with 5 × 10^6^/0.1 ml of various MCF7/ADR cell lines. Bi-dimensional tumor measurements were taken every two days. Tumor volume (mm^3^) was calculated as follows: V = 1/2L×W^2^ (L: length, W: width). The mice were treated by DOX (4 mg/kg/week) or PBS by intraperitoneal injection. The nude mice were euthanized using CO_2_ overdose, tumor xenografts were removed, and the specimens were fixed with neutral-buffered 10% formalin and embedded in paraffin blocks.

### Immunohistochemical staining

Section (4 µM) of tumor blocks were used for immunohistochemical analysis. Sections were treated with primary antibodies. The slides were stained bydiaminobenzedine solution (DAB), and then counterstained with haematoxylin. The slices were photographed by a Leica Microscope and Image Pro Plus analyse system. Four high-power views (400×) were selected randomly from each sample in a blinded manner; the level of integrated OD was estimated and presented as mean ± SEM.

### Dual-luciferase reporter assay

The dual-luciferase reporter assay system was obtained from Promega Corporation (Madison, WI, USA). The 3’-UTR-luciferase reporter constructs containing the 3’-UTR region of KIF2C with the wild-type and mutant binding sites of miR-152, as well as the promotor region of COPZ2 (miR-152 host gene) with the binding sites of TBX15, were amplified by PCR. All constructs containing inserts of the 3’-UTR region were sequenced and validated. Luciferase activities were measured 24 h after transfection using the Dual Luciferase Reporter Assay System. Experiments were performed with three independent replicates.

### Statistical analysis

The co-expression analysis between miR-152 and the intersection genes was used by the Spearman’s correlation analysis method. The overall survival (OS) of TBX15 or KIF2C was analyzed by the Kaplan Meier plotter in The Cancer Genome Atlas (TCGA) database.

Other data are presented as mean ± SEM. P-values were calculated by one-way ANOVA or Student’s t-test for unpaired samples using the GraphPad Prism software. The results were considered significant at P < 0.05.

## Results

### TBX15 overexpression abrogates breast cancer DOX resistance by suppressing glycolysis and autophagy

**Fig. 1:**
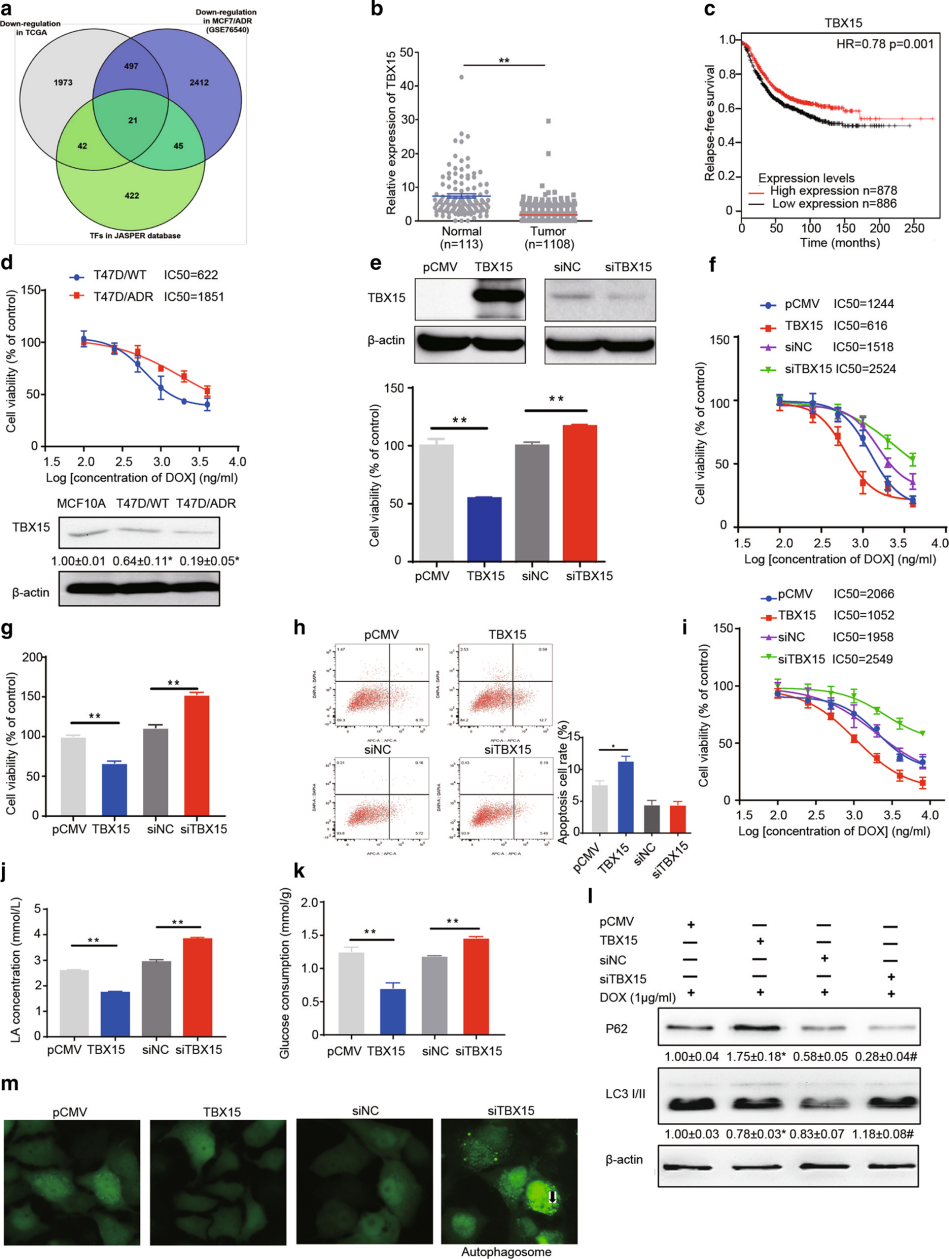
TBX15 reduced DOX resistance in breast cancer. **A** Venn diagram showing the intersection of genes identified in TCGA that are downregulated in breast cancer, genes downregulated in MCF7/ADR cells (GSE76540 database), and from TFs identified in the JASPER database. **B** Relative gene expression of TBX15 in normal tissue or breast tumors. Gene expression data were obtained from 1108 breast tumors and 113 normal adjacent tissues from TCGA dataset and were presented as the means ± SEM. **C** Kaplan–Meier plot predicting the Relapse-Free Survival of patients with high or low expression of TBX15. **D** T47D/ADR cells were developed from T47D/WT cells, and the IC50 values of DOX treatment were tested. TBX15 expression in MCF10A, T47D/WT and T47D/ADR cells was detected in Western blotting assays. β-actin was used as internal reference. **E**–**N** Breast cancer cells were transfected with pCMV, pCMV-TBX15, siNC, or siTBX15. **E** The T47D/ADR cells with different treatments were sent to MTT assays. **F** Drug sensitivity analysis after DOX treatment in various T47D/ADR groups. **G** The MCF7/ADR cells with various treatments were sent to MTT assay. **H** The MCF7/ADR cells with various treatments were sent to flow cytometry to test cell apoptosis rate. **I** Drug sensitivity analysis after DOX treatment in MCF7/ADR cells. **J–K** Lactate acid (LA) production and glucose consumption were detected in various T47D/ADR groups. **L** P62 and LC3 II/I expression in MCF7/ADR cells were detected in Western blotting assays. β-actin was used as internal reference. **M** The MCF7/ADR cells were transfected with EGFP-LC3 plasmid, and then exhibited various treatments. Fluorescence microscope was used to investigate the autophagy process (400×magnification, Arrows: autophagosomes accumulated with LC3). Data are presented as the means± SEM from three independent experiments. *, **P < 0.05, *P* < 0.01, respectively. ^#^ indicates significant difference compared to siNC group at *P* < 0.05

To identify the mechanisms of DOX resistance in breast cancer cells, using bioinformatics methods we found potential 21 transcription factors (TFs) (ZEB1, TEAD1, NFAT5, MEOX2, TWST1, MAFF, RFX2, MEF2C, EGR2, EBF1, KLF14, JUN, MEIS2, NR3C2, BACH2, BHLHE41, NR3C1, TBX15, MSX1, LHX6, ETV5) that were downregulated in both breast cancer tissues and DOX-resistant breast cancer cells in the JASPER database (Fig. 1A). To test whether expression levels of these TFs were downregulated in a large and diverse breast cancer cohort, we analyzed their expression levels of these factors in the cancer tissues and in adjacent normal tissues. Among them, TBX15 levels were downregulated in breast cancer tissues, compared to normal adjacent tissues (Fig. 1B), and acted as a positive marker for the Relapse-Free survival prognosis of breast cancer (Fig. 1C). We induced T47D DOX-resistant breast cancer cells (T47D/ADR) by long exposure of DOX treatment in the cells, then detected its TBX15 expression level. Figure 1D showed that TBX15 protein levels were decreased in T47D/ADR cells. To further investigate the effects of TBX15 expression on DOX sensitivity, we utilized two DOX-resistant breast cancer cell lines, MCF7/ADR, and T47D/ADR, and treated these cells with varying concentrations of DOX. Following that, the cell viability assays and drug sensitivity analysis showed that TBX15 overexpression caused a significant reduction in cell growth and IC50 values; siRNAs targeting TBX15 (siTBX15) increased cell growth and IC50 values (Fig. 1E–I).

Activation of autophagy and glycolysis were reported to be important in drug resistance in various cancer cells [24, 25]. Additionally, TBX15 expression was associated with glycolysis in some tissues [26]. In order to determine mechanisms by which TBX15 may effects DOX resistance, we transfected TBX15 plasmid or TBX15 siRNAs into DOX-resistant cells, and assessed the expression levels of markers of autophagy or glycolysis. TBX15 overexpression decreased glycolysis by glucose and lactate assays, whereas siTBX15 increased glycolysis (Fig. 1J, K). Furthermore, TBX15 overexpression increased p62 protein expression and suppressed recruitment and conversion of LC3-I to LC3-II. In contrast, siTBX15 decreased p62 protein expression and promoted LC3-I conversion (Fig. 1L). At the same time, siTBX15 transfection promoted autophagosome formation after DOX treatment (Fig. 1M). These data indicate that TBX15 overexpression abrogates DOX resistance by inhibiting autophagy and glycolysis in breast cancer cells.

### TBX15 forced expression promotes the DOX sensibility by inducing miR-152 expression

**Fig. 2:**
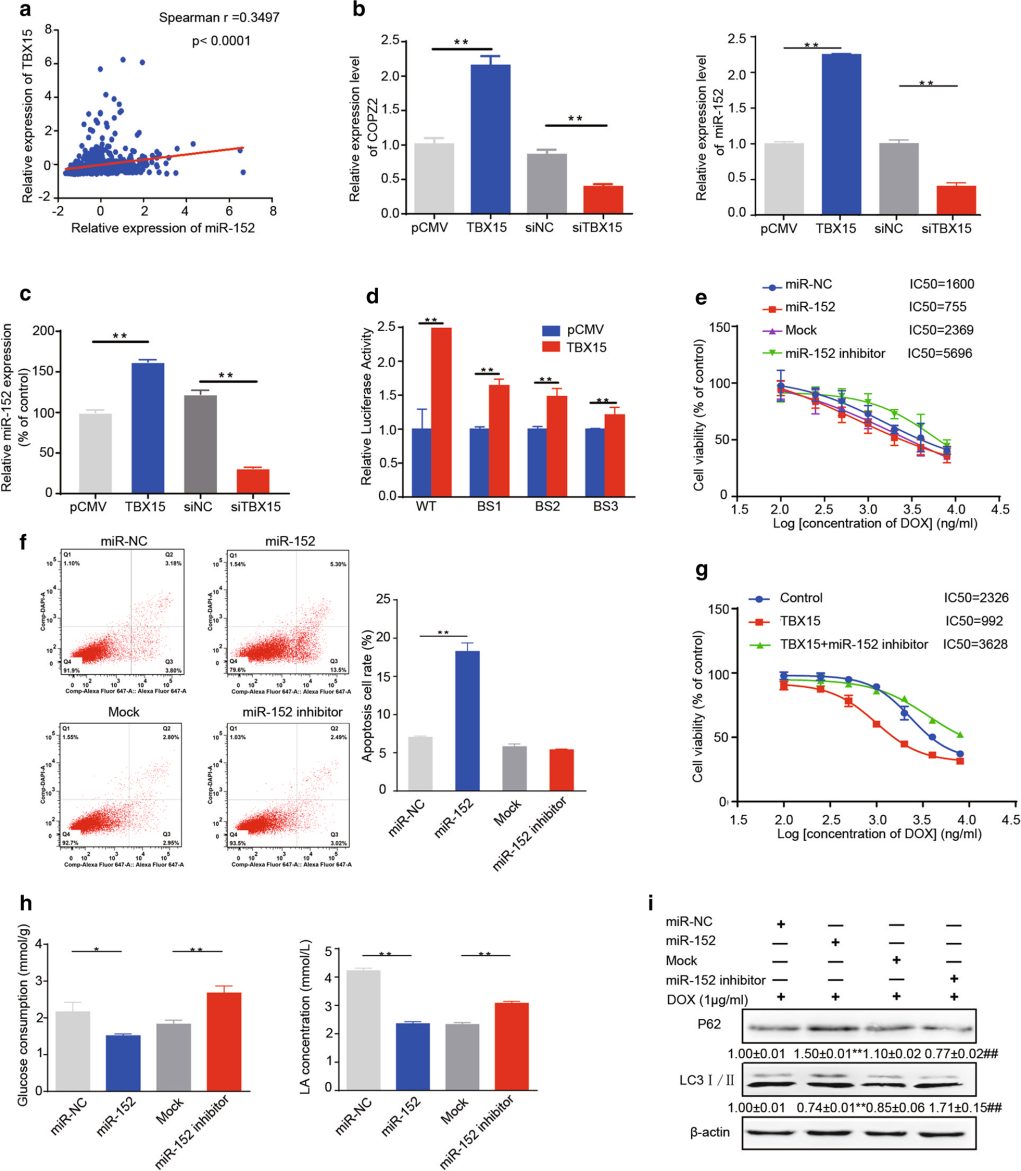
TBX15 inhibited DOX resistance in breast cancer through inducing miR-152 expression. **A** Spearman’s correlation analysis of TBX15 and miR-152 expression in TCGA dataset. **B** MCF7/ADR cells were treated with TBX15 overexpression or siTBX15, and the expression levels of miR-152 and its host gene were analyzed by qRT-PCR. **C** T47D/ADR cells were treated with TBX15 overexpression or siTBX15, and miR-152 expression level was analyzed by qRT-PCR. **D** The luciferase activity was analyzed using Promega Dual-Luciferase Reporter Assay System. The wild type of miR-152 promoter region is located in-320–1800 bp. Three TBX15 binding site sequences: BS1 is located in-322–381 bp, BS2 in -1017–1212 bp, and BS3 in-1536–1793 bp. Three TBX15 binding site sequences in the miR-152 promoter region were cloned into the luciferase reporter vectors. HEK293T cells were co-transfected with TBX15 expression vector (WT) or mutative vectors and a renilla luciferase vector. **E** MCF7/ADR Cells transfected with miR-152 mimics or miR-152 inhibitor were treated with DOX for 48 h, and subsequently added with MTT and obtain IC50 values. **F** Flow cytometric plots of MCF7/ADR cells that were transfected as in **E** and were treated with 1 µg/ml DOX. Cells were harvested after 48 h to assess apoptosis. **G** Drug sensitivity was analyzed from the cells that were treated with TBX15 overexpression and miR-152 inhibitor. **H** Glucose consumption (left) and lactate acid concentrations in the medium (right) were tested in MCF7/ADR cells transfected as in **E**. **I** Expression levels of LC3II/I and p62 were analyzed by Western blotting with β-actin used as an internal control in cells transfected as in **E**. For all panels, data are presented as the means ± SEM from three independent experiments. ** Indicates significant difference compared to miR-NC group at *P* <0.01. ^##^ indicates significant difference compared to mock group at *P* < 0.01

We found that levels of TBX15 and miR-152 strongly correlated in TCGA database using Spearmen’s correlation analysis (Fig. 2 A). Forced expression of TBX15 increased miR-152 expression levels in MCF7/ADR and T47D/ADR cells, while knockdown of TBX15 decreased miR-152 expression (Fig. 2B, C). These data showed TBX15 upregulated miR-152 expression levels. To verify whether TBX15 promoted miR-152 expression through transcriptional regulation, we first used the JASPER database and identified three binding sites of TBX15 in the miR-152 promoter region. Subsequently, we constructed reporter plasmids and performed luciferase promoter reporter assays to determine whether TBX15 upregulated miR-152 expression by binding to miR-152 promoter region. These data showed that overexpression of TBX15 induced luciferase activities at all three binding sites we predicted (Fig. 2D).

To test whether miR-152 suppresses DOX resistance in breast cancer tissue, we analyzed miR-152 expression in the cancer tissues and adjacent tissues in TCGA database. Breast cancer tissues had lower expression levels of miR-152 when compared to the adjacent tissue group (Additional file 1: Fig. S1A). Furthermore, we subdivided the TCGA dataset into miR-152 high and miR-152 low group according to its expression levels and sent them to gene set enrichment analysis (GSEA) which showed that lower expression levels of miR-152 were enriched in KANG DOXORUBINCIN UP dataset (NES = 1.56, FDR q = 0.01). These data indicated the strong correlation between miR-152 attenuation and DOX resistance in breast cancer (Additional file 1: Fig. S1B).

We investigated the mechanisms by which miR-152 may affect DOX resistance. The cells overexpressing miR-152 were more sensitive to DOX treatment, as indicated by a lower IC50 value and higher apoptosis rate. Correspondingly, the cells in which miR-152 was inhibited displayed an increased resistance to DOX treatment (Fig. 2E, F). On the contrary, miR-152 inhibitor reversed TBX15-suppressed DOX resistance in breast cancer cells (Fig. 2G).

To examine the mechanisms of miR-152 on these processes, we transfected miR-152 mimics or a miR-152 inhibitor into MCF7/ADR cells and assessed its effects on autophagy or glycolysis. miR-152 mimics decreased glycolysis in glucose and lactate assays, whereas miR-152 inhibitor increased glycolysis (Fig. 2H). Furthermore, miR-152 mimics increased p62 protein expression and suppressed recruitment and conversion of LC3- I to LC3- II. In contrast, miR-152 inhibitor decreased p62 protein expression and promoted LC3-I conversion (Fig. 2I). These data indicate that TBX15/miR-152 pathway abrogates DOX resistance by inhibiting autophagy and glycolysis in breast cancer cells. TBX15 attenuation may result in miR-152 downregulation in DOX-resistant breast cancer cells, suggesting a potential new therapeutic target for breast cancer diagnosis and treatment in the future.

### KIF2C, a direct target of miR-152, is upregulated in breast cancer which induces glycolysis and autophagy

**Fig. 3:**
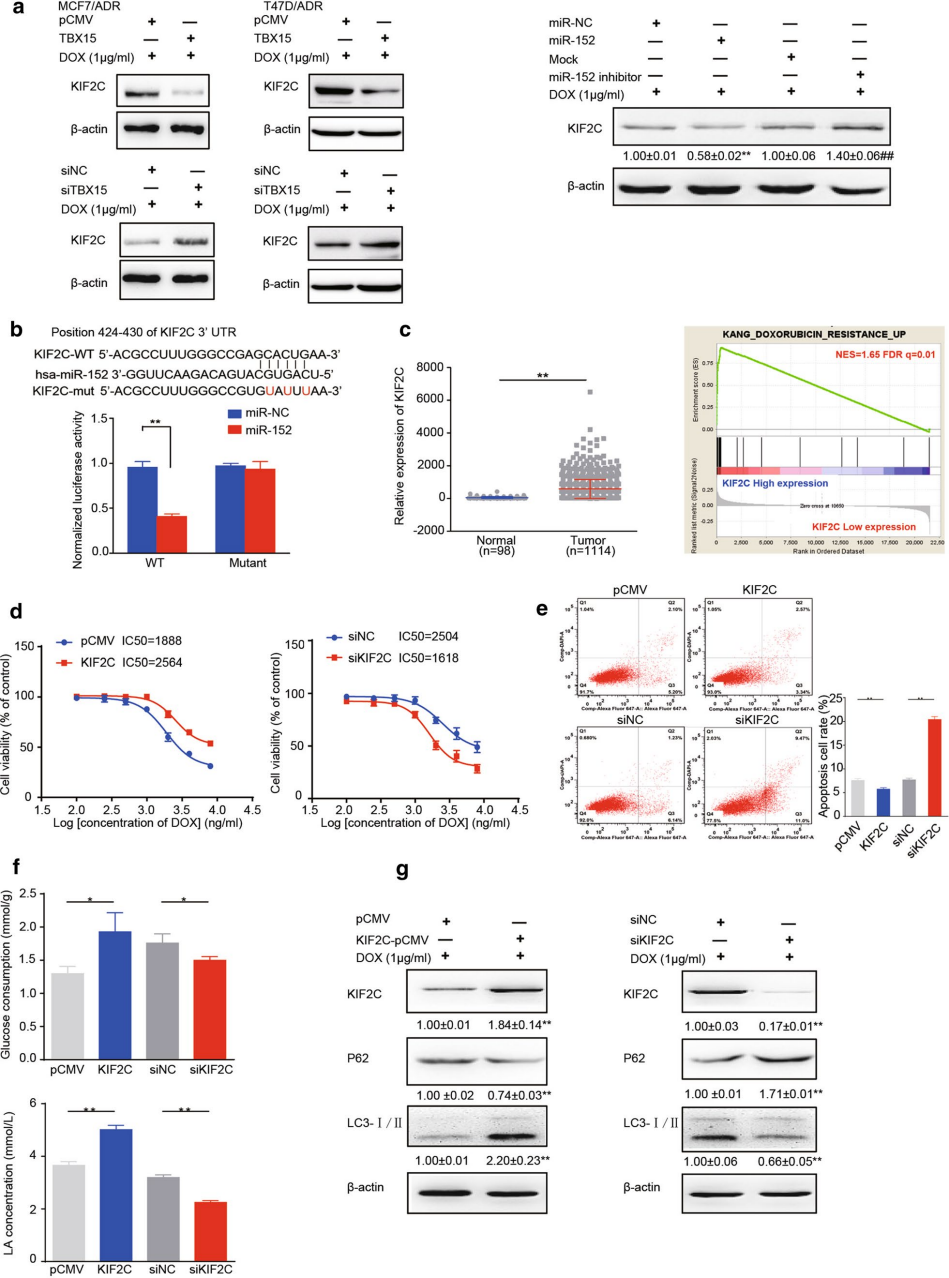
TBX15/miR-152 pathway directly targets and inhibits KIF2C. **A** Western blotting analysis for protein expression of KIF2C in MCF7/ADR cells and T47D/ADR cells transfected with miR-152 mimics or miR-152 inhibitor, or TBX15-pCMV or siTBX15. All cell groups were treated with 1 µg/ml DOX. **B** Putative seed-matching sites or mutant sites (red) between miR-152 and the 3’-UTR of KIF2C were analyzed by TargetScan. The WT and mutant (mut) binding site sequences of miR-152 in the 3’ UTR of KIF2C were cloned into luciferase reporter vectors. HEK293T cells were co-transfected with the reporter vectors, renilla luciferase vector, and miR-152 mimics or miR-NC. After 24 h, the relative levels of luciferase activity were analyzed and normalized to values obtained from WT cells transfected with miR-NC. **C** Relative expression levels of KIF2C from normal tissue and breast tumors from human patients. KIF2C mRNA expression data were obtained from TCGA database containing 1114 breast tumors and 98 normal adjacent tissues, and were presented as the means ± SEM from three independent experiments. GSEA analysis to evaluate the correlation between expression levels of KIF2C and DOX resistance signatures in the GEO database. **D** MCF7/ADR cells were transfected with pCMV or KIF2C-pCMV plasmids (KIF2C), siNC or siKIF2C and were treated with DOX. After 48 h, drug sensitivity analysis was determined. **E** Flow cytometric analysis of MCF7/ADR cells which were transfected as in **D** and treated with 1 µg/ml DOX. **F**, **G** MCF7/ADR cells were transfected as above and were treated with 1 µg/ml DOX. After 48 h, the glucose consumption (**F**, top), LA production (**F**, bottom), and protein expression levels of KIF2C, p62, LC3, β-actin (**G**) were analyzed. All the data are presented as the means±SEM from three independent experiments and were normalized with corresponding control. *, ** *P* < 0.05 and *P* < 0.01, respectively

KIF2C is upregulated in some cancer tissues and is associated with resistance to chemotherapy [15–17]. By bioinformatic assays, we found KIF2C is one of potential target of miR-152. We analyzed the correlation between expression levels of miR-152 and its potential targets, and found that KIF2C was one of most significantly positive-associated gene with miR-152 expression levels, and miR-152 levels were negatively correlated with KIF2C expression in breast cancer in the GEO database (Additional file 2: Fig. S2A, B). DOX-resistant cells transduced with TBX15 or miR-152 mimics have lower KIF2C expression levels following DOX treatment, whereas transfection with siTBX15 or miR-152 inhibitor increased KIF2C expression levels in both MCF7/ADR and T47D/ADR cells (Fig. 3A). By using bioinformatic analysis of miR-152 seed-matching site in the KIF2C gene, we found that KIF2C was a potential direct target of miR-152. To confirm this, luciferase reporter constructs were made that contained the putative binding site of the KIF2C 3’-UTR region or three nucleotide substitutions in its 3’-UTR region (mutant). Overexpression of miR-152 inhibited wild-type KIF2C reporter activity, but not that mutant region (Fig. 3B), demonstrating that miR-152 can specifically target the 3’-UTR regions of KIF2C by binding to its putative sequences.

To identify whether KIF2C promotes DOX resistance in breast cancer tissues, we obtained TCGA mRNAs expression data from Breast Cancer, and divided the samples into cancer tissues and adjacent tissue groups. We found that KIF2C expression levels were significantly higher in breast cancer tissues than those in the adjacent groups (Fig. 3C). We then divided the cancer samples into groups based on high or low expression levels of KIF2C, and assessed overall patient outcomes within these two groups. Higher expression of KIF2C indicated poor prognosis in breast cancer patients (Additional file 2: Fig. S2C). In addition, we also observed the positive correlation between KIF2C expression and DOX resistance signature using the GSEA (NES = 1.65, FDR q = 0.01) which suggesting high expression levels of KIF2C may be responsible for DOX resistance (Fig. 3C).

Additionally, we determined the IC50 value and performed cell apoptosis assays to detect DOX sensitivity in KIF2C-upregulated or down-regulated MCF7/ADR cells. Cells overexpressing KIF2C were more sensitive to DOX treatment, while those transfected with siKIF2C displayed an increased resistance to DOX (Fig. 3D). Similarly, apoptosis assays showed KIF2C overexpression suppressed cell apoptosis after DOX treatment, while siKIF2C displayed an increased sensitivity to DOX (Fig. 3E). To identify the mechanism of KIF2C-mediated DOX resistance, overexpressed or inhibited expression of KIF2C in MCF7/ADR cells were used to assess autophagy and glycolysis. Overexpression of KIF2C increased glycolysis in glucose and lactate assays, whereas siKIF2C reduced glycolysis (Fig. 3F). Overexpression of KIF2C decreased p62 protein expression and promoted conversion of LC3-I, whereas siKIF2C increased p62 protein expression and suppressed LC3-I conversion (Fig. 3G). Collectively, these data suggest that KIF2C promotes DOX resistance by inducing autophagy and glycolysis.

**Fig. 4:**
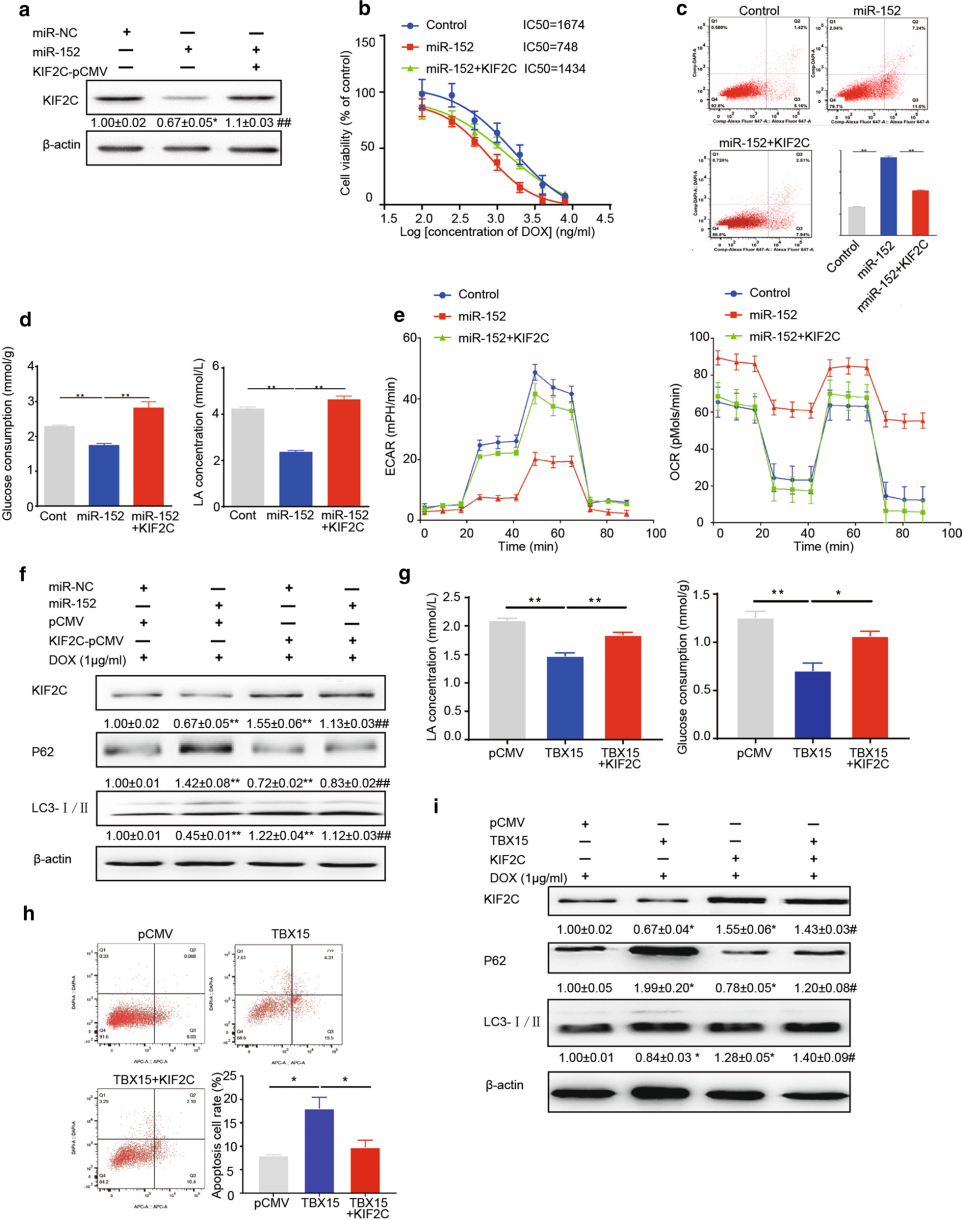
KIF2C overexpression reverses miR-152-induced DOX sensitivity in MCF7/ADR cells. **A** Levels of KIF2C in MCF7/ADR cells which were co-transfected with miR-NC, miR-152 mimics, or KIF2C. The data are presented as the means±SEM from three independent experiments. * Compared to miR-NC group at *P* < 0.05, ^##^ compared to miR-152 at *P * <  0.01. **B** IC50 values of MCF7/ADR cells that were transfected as in **A** and were treated with DOX and cultured for 48 h. **C** Flow cytometric analysis of MCF7/ADR cells that were transfected as in (A) and were treated with 1 µg/ml DOX. **D**, **E** The glucose consumption (**D**, left), LA production (**D**, right), ECAR) (**E**, left) and OCR (**E**, right) were analyzed in MCF7/ADR cells treated as in **A**. **F** Levels of KIF2C, p62 and LC3 in MCF7/ADR cells treated as in (**A**). Graph represents the relative density of KIF2C, p62, or LC3-II/LC3-I expression. The data are presented as the means ± SEM from three independent experiments. ** Compared to miR-NC group at *P* < 0.01, ^##^ compared to miR-152 at *P *< 0.01. **G** The glucose consumption (**G**, top) and LA production (**G**, bottom) were analyzed in MCF7/ADR cells treated with TBX15 or KIF2C overexpression. **H** Flow cytometric analysis of MCF7/ADR cells that were transfected as in **G** and were treated with 1 µg/ml DOX. **I** Levels of p62 and LC3 in MCF7/ADR cells treated as in **G**. Graph represents the relative density of KIF2C, p62, or LC3-II/LC3-I expression. The data are presented as the means ± SEM from three independent experiments. *, ** Compared to control groups at *P* < 0.05 and *P* < 0.01, ^#^ compared to TBX15 at *P * < 0.05

To determine role of KIF2C in TBX15/miR-152-mediated drug sensitivity, we tested the effects of KIF2C on the DOX sensitivity by transfecting MCF7/ADR cells overexpressing TBX15/miR-152 with KIF2C cDNA (without the 3’-UTR region). Overexpression of KIF2C suppressed miR-152-induced DOX sensitivity, as determined by IC50 values and apoptosis assays (Fig. 4A–C). The reduction of glucose consumption, lactate production, ECAR and OCR assays induced by miR-152 overexpression was abrogated in these cells (Fig. 4D, E). Furtherly, KIF2C overexpression reversed miR-152-suppressed autophagy (Fig. 4F). Similarly, KIF2C suppressed TBX15-induced DOX sensitivity by increasing autophagy and glycolysis (Fig. 4G–I). Taken together, these data suggest that TBX15/miR-152 pathway promotes DOX sensitivity through the targeting of KIF2C by miR-152.

### KIF2C increased PKM2 expression by promoting its protein stability

**Fig. 5:**
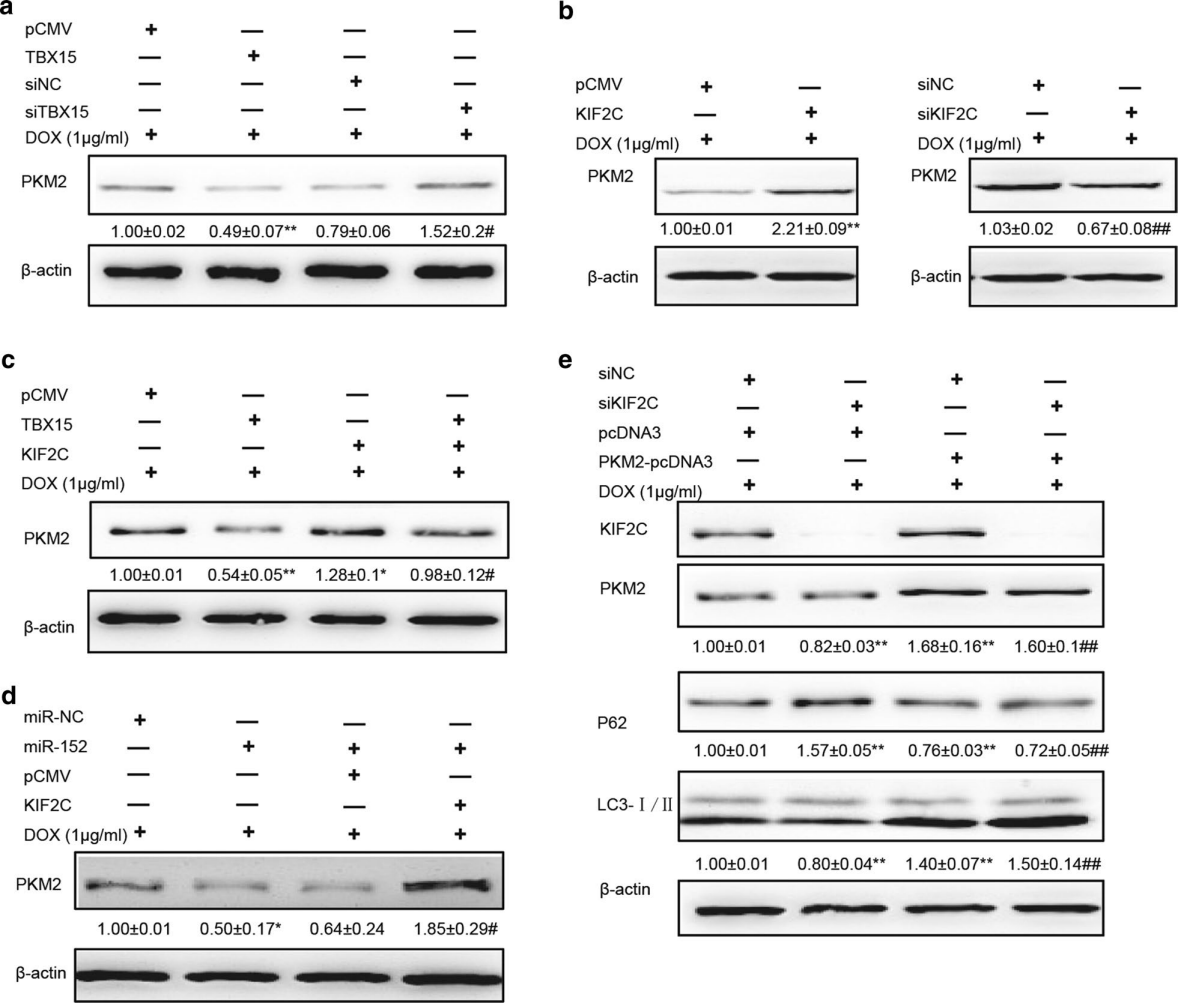
PKM2 is involved in DOX resistance in breast cancer, mediated by the miR-152/KIF2C signaling pathway. **A** Western blotting analysis of PKM2 levels in MCF7/ADR cells that were transfected with pCMV, TBX15, siNC or siTBX15, and were treated with 1 µg/ml DOX for 48 h. β-actin served as an internal control. **B** Western blotting analysis of PKM2 levels in MCF7/ADR cells transfected with KIF2C or siKIF2C, and were treated with 1 µg/ml DOX for 48 h. **A**, **B** Graph represents the relative density of PKM2 expression. β-actin was used as an internal control. The data are presented as the means ± SEM from three independent experiments.** Indicates significant difference compared to pCMV group at *P* < 0.01, ^#^,^##^ compared to siNC at *P *< 0.05, *P *< 0.01, respectively. **C** Western blotting analysis of PKM2 levels in MCF7/ADR cells transfected with TBX15 and /or KIF2C, and were treated with 1 µg/ml DOX for 48 h. β-actin was used as an internal control. Graph represents the relative density of PKM2 expression. *,** Indicate significant difference compared to pCMV group at *P *< 0.05 and *P* < 0.01, respectively. ^#^ Compared to TBX15 at *P *< 0.05. **D** Western blotting analysis of PKM2 in MCF7/ADR cells that were co-transfected with miR-NC, miR-152 or KIF2C and were treated with 1 µg/ml DOX for 48 h. β-actin was used as an internal control. * Indicates significant difference compared to miR-NC group at *P *< 0.05, ^#^ compared to miR-152 at *P *< 0.05. **E** Western blotting analysis of KIF2C and PKM2 in MCF7/ADR cells that were co-transfected with siNC, siKIF2C or PKM2-pcDNA, and were treated with 1 µg/ml DOX for 48 h. Graph represents the relative density levels of PKM2, p62, or LC3-II/ I expression. β-actin was used as an internal control. ** Indicates significant difference compared to siNC group at *P *< 0.01, ^##^ compared to siKIF2C at *P *< 0.01

PKM2 is involved in multidrug resistance in some cancer cells [22, 27]. However, role of PKM2 in mediating chemoresistance in breast cancer remains to be studied. In our previous study, we found that PKM2 promoted cell proliferation and angiogenesis in breast cancer [12]. In order to identify whether PKM2 is involved in the TBX15/miR-152/KIF2C-mediated DOX resistance, we assessed PKM2 expression in MCF7/ADR cells when expression of TBX15/miR-152 or KIF2C was altered in the cells. TBX15 overexpression decreased PKM2 expression, whereas its siRNAs increased PKM2 levels (Fig. 5A). Conversely, overexpressing KIF2C increased PKM2 expression, whereas knockdown of KIF2C caused its expression decrease (Fig. 5B). Notably, overexpression of KIF2C reversed TBX15 or miR-152-mediated downregulation of PKM2 (Fig. 5C, D). In addition, PKM2 overexpression restored siKIF2C-inhibited autophagy (Fig. 5E). Thus, these data suggest that PKM2 is regulated in TBX15/miR-152/KIF2C pathway for regulating DOX resistance.

**Fig. 6:**
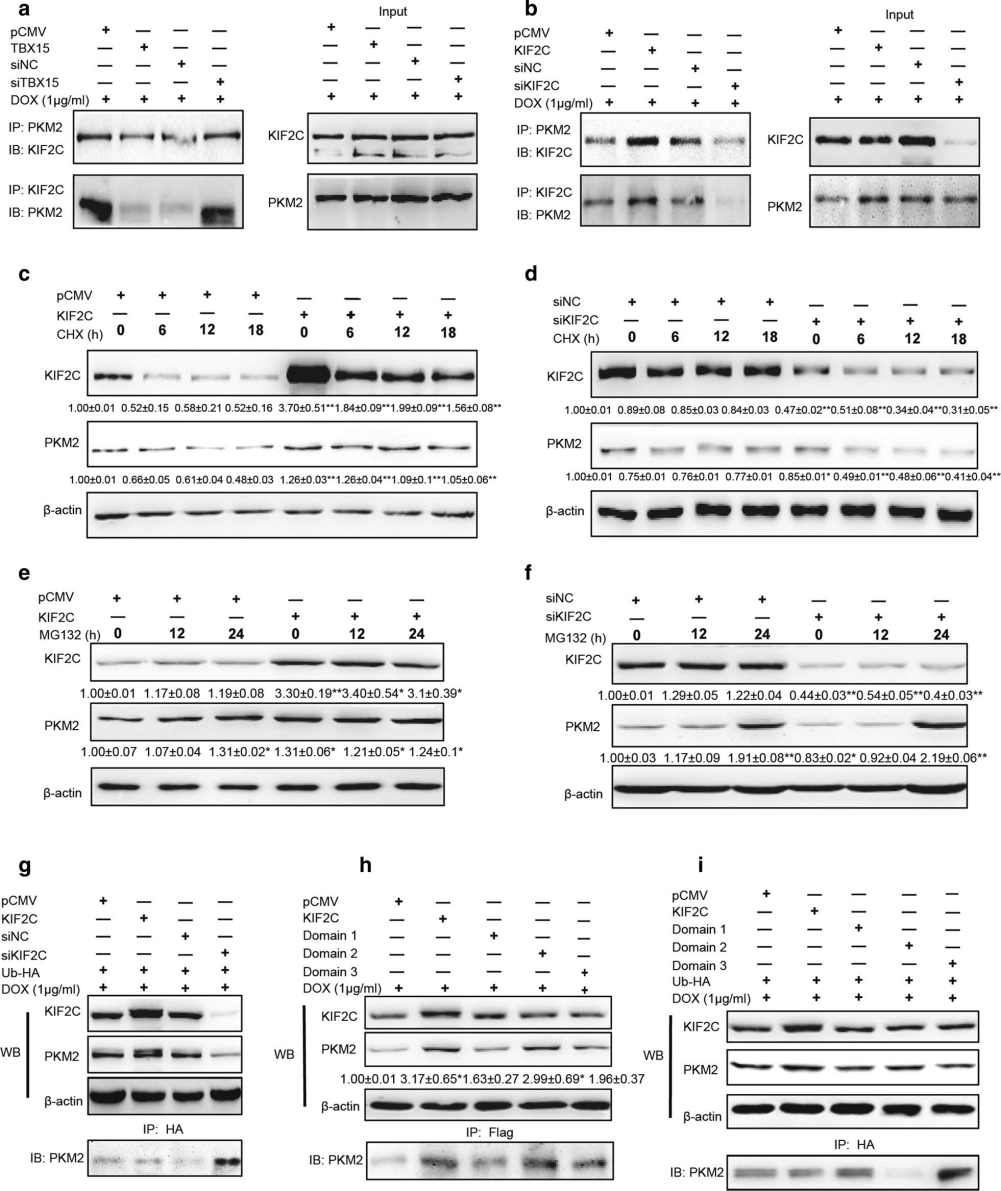
KIF2C directly binds to PKM2 and promotes PKM2 stability in breast cancer cells. **A** Co-IP analysis of MCF7/ADR cells that were transfected with pCMV, TBX15, siNC or siTBX15 and were treated with 1 µg /ml DOX for 48 h. **B** Co-IP analysis of MCF7/ADR cells that were transfected with pCMV, KIF2C, siNC or siKIF2C. Cell lysates were co-immunoprecipitated using an anti-KIF2C or anti-PKM2 antibody. **C** Western blotting analysis of MCF7/ADR cells transfected with pCMV or KIF2C and treated with 20 µg /ml CHX for 0, 6, 12 and 18 h. **D** Western blotting analysis of MCF7/ADR cells transfected with siNC or siKIF2C and were treated with CHX. The expression levels of KIF2C and PKM2 were analyzed. **E** Western blotting analysis of MCF7/ADR cells transfected with a pCMV or KIF2C and treated with 1 µM MG132 for 0, 12 and 24 h. **F** Western blotting analysis of MCF7/ADR cells transfected with siNC or siKIF2C and treated with MG132. The expression of KIF2C and PKM2 were analyzed. **G** Western blotting analysis of MCF7/ADR cells transfected with pCMV, KIF2C, siNC or siKIF2C and Ubiquitin-HA plasmid. Cell lysates were co-immunoprecipitated using anti-HA antibody and the expression levels of Ub-PKM2 were analyzed. **H** Western blotting analysis of MCF7/ADR cells transfected with a pCMV, KIF2C, and KIF2C domains (D1, D2, D3) in pCMV-flag plasmids. Cells were treated with 1 µg /ml DOX for 48 h. Cell lysates were co-immunoprecipitated using anti-flag antibody and the expression levels of PKM2 were analyzed. **I** Western blotting analysis of MCF7/ADR cells that were transfected as (**H**), and Ubiquitin-HA plasmid. Cell lysates were co-immunoprecipitated using an anti-HA antibody and the expression levels of Ub-PKM2 were analyzed. All the data above were presented as the means ± SEM from three independent experiments and were normalized to the percentage of corresponding control groups. *, ** *P* < 0.05, *P* < 0.01, respectively

In order to determine the mechanism by which KIF2C regulates PKM2 expression, mRNA expression levels of PKM2 were determined in MCF7/ADR cells after the transfection of KIF2C. Overexpression of KIF2C did not change PKM2 mRNA expression levels (data not shown), suggesting a potential posttranscriptional mechanism modulated PKM2 expression. Co-IP analysis showed that KIF2C directly bound to PKM2, and that a weaker PKM2 band was co-immunoprecipitated by anti-KIF2C antibodies when TBX15 was overexpressed. In contrast, transfection of siTBX15 resulted in a stronger interaction between KIF2C and PKM2 (Fig. 6A). Similarly, KIF2C overexpression also resulted in a stronger interaction between KIF2C and PKM2; and siKIF2C decreased the binding (Fig. 6B).

To determine whether KIF2C modulates PKM2 protein stability in MCF7/ADR cells, we treated cells with CHX, which inhibits protein production. CHX treatment did not change KIF2C-mediated upregulation of PKM2 (Fig. 6C). However, knockdown of KIF2C enhanced PKM2 degradation following CHX treatment in a time dependent manner (Fig. 6D). We also treated cells with MG132, which inhibited protein degradation. It restored the downregulation of PKM2 following knockdown of KIF2C (Fig. 6E, F). Considering the ubiquitin (Ub)-editing degradation of PKM2, we examined the ubiquitylation pattern of PKM2 in MCF7/ADR cells. Ub-HA plasmid was co-transfected with KIF2C-pCMV or siKIF2C. Co-immunoprecipitation experiments revealed that overexpression of KIF2C induced weaker bands, which corresponded to PKM2 ubiquitination, whereas knockdown of KIF2C enhanced PKM2 ubiquitination (Fig. 6G). Therefore, these data suggest that KIF2C directly binds to PKM2 and prevent PKM2 degradation by ubiquitination.

There are three domains (Domain-1, Domain-2, and Domain-3) of human KIF2C protein (Additional file 2: Fig. S2D, http://www.uniprot.org/uniprot/Q99661). To confirm which domain(s) was/were responsible for PKM2 ubiquitylation, we constructed three plasmids encoding different domains of KIF2C, respectively. The three plasmids were transfected into MCF7/ADR cells, respectively, and PKM2 expression levels were analyzed by Western blotting. Only overexpression of the plasmid encoding Domain-2 of KIF2C increased PKM2 expression and showed more binding to PKM2 in co-immunoprecipitation experiments (Fig. 6H). Ubiquitin immunoprecipitation assays also revealed that only overexpression of KIF2C Domain-2 resulted in a weak band that corresponded to PKM2 ubiquitination (Fig. 6I). These data suggest that KIF2C Domain-2 plays a major role in the binding of KIF2C and PKM2 to increase PKM2 stability.

### TBX15/miR-152 overexpression abrogates DOX resistance in vivo

**Fig. 7:**
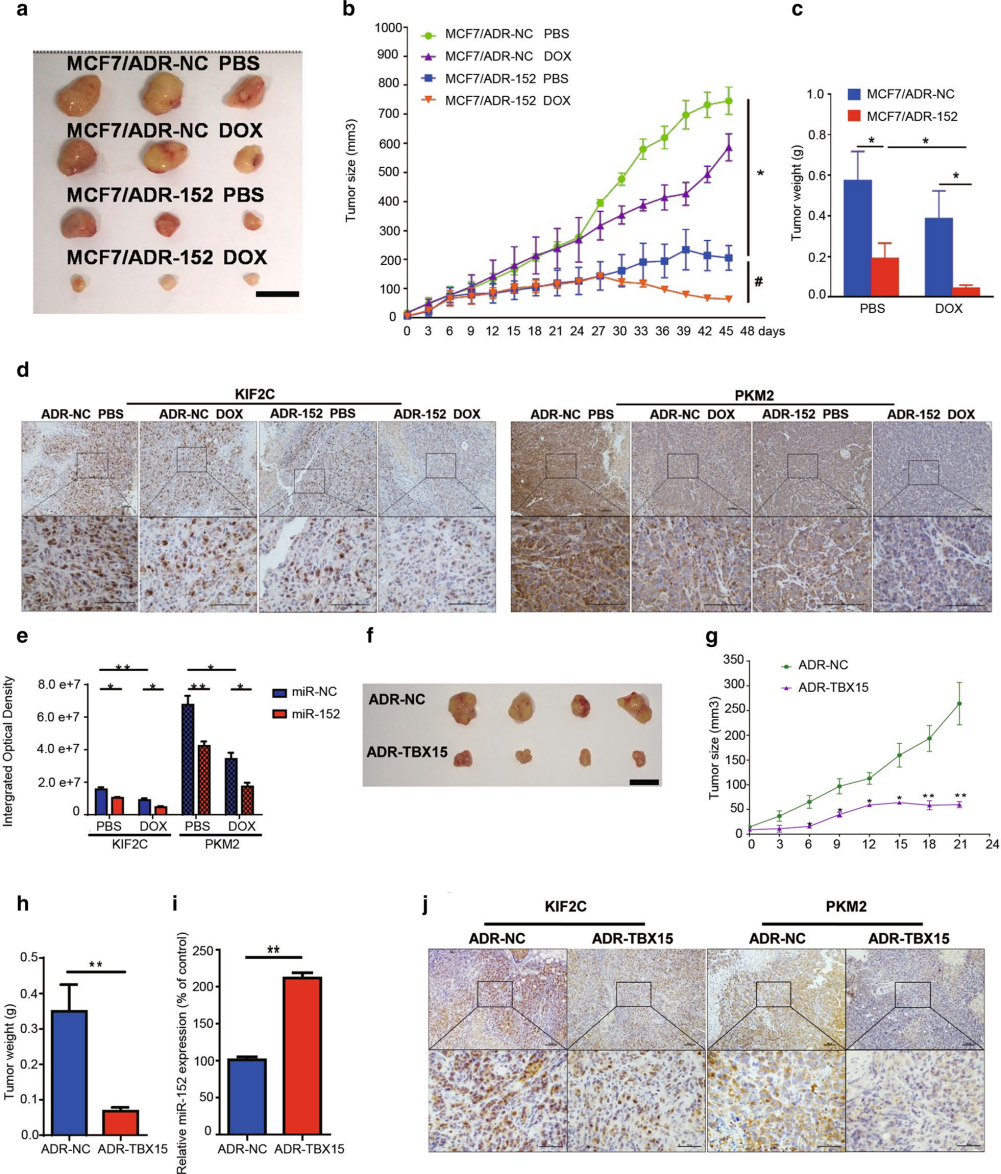
TBX15/miR-152 overexpression reverses DOX resistance in vivo by decreasing KIF2C and PKM2 expression. **A–C** The MCF7/ADR cells expressing miR-152 or a control (miR-NC) were established using a lentivirus system and dispersed in 100 µl of serum-free DMEM medium. Cells were subcutaneously injected into female nude mice (n = 8). After subcutaneous injection of cancer cells for 24 days, mice were treated with DOX or PBS. Representative pictures of tumors (**A**), tumor size (**B**), and weight (**C**) were obtained at the indicated time point or following 45 days after initial tumor cell injection. Tumors were measured every two days and the tumor volumes were calculated. The tumors were excised and weighed after 45 days, and the representative pictures of trimmed tumors are displayed (Bar= 10 mm). Data are presented as the means ± SEM from all tumor samples. **D**, **E** Immunohistochemistry staining of tumor tissues with antibodies of KIF2C and PKM2 (bar = 100 μm). * *P* < 0.05, ** P < 0.01. **F**, **J** The MCF7/ADR cells overexpressing TBX15 or a Negative control (NC) were established using lentivirus system. Cells were subcutaneously injected into female nude mice (n = 8). 10 days after cancer cells subcutaneous injection, tumor xenografts were found in ADR-NC group. However, only 4 tumor samples were found 31 days after injection in TBX15 overexpression group (**F**). Tumors growth was measured as above. The tumors were excised and weighed, and the representative pictures of trimmed tumors were displayed (Bar= 10mm). Data were presented as the means ± SEM from all tumor samples (**G**, **H**). **I** qRT-PCR analysis of miR-152 gene expression in ADR-NC and ADR-TBX15 tumor tissues. Data were normalized to the ratio of miR-152 in ADR-NC group. Data are presented as the means± SEM from all tumor samples. **P  < 0.01. **J** Immunohistochemistry staining of tumor tissues with antibodies of KIF2C and PKM2 (Bar= 100 μm in Low Power view and Bar= 50 μm in High Power view)

In order to investigate the effects of miR-152 on DOX sensitivity in vivo, we established tumor xenografts by subcutaneously implanting MCF7/ADR cells that were stably expressing miR-152 in nude mice. Twenty-four days after subcutaneous injection of the cancer cells, the mice were treated with DOX (4 mg/kg/week) and or PBS by intraperitoneal injection. Analysis of implanted tumor xenografts revealed that overexpression of miR-152 significantly suppressed tumor growth. Furthermore, xenografts with overexpression of miR-152 were more sensitive to DOX treatment (Fig. 7A–C). These data suggested that miR-152 promotes sensitivity to DOX treatment in vivo. Using immunohistochemistry assays, we found that overexpression of miR-152 significantly suppressed KIF2C and PKM2 expression, and that this suppression was much greater following DOX treatment (Fig. 7D, E). Collectively, these data suggest that miR-152 abrogates DOX resistance by inhibiting KIF2C and PKM2 expression.

In order to elucidate the effect of TBX15 on miR-152, KIF2C and PKM2 expression in vivo, we established tumor xenografts by implanting MCF7/ADR cells stably expressing TBX15. The tumors were found 10 days after subcutaneous injection. The mice were euthanized 21 days after the cell injection, and tumors were resected. Analysis of tumor tissues revealed that overexpression of TBX15 significantly decreased tumor growth (Fig. 7F–H). Furthermore, qRT-PCR and immunohistochemistry assays showed that TBX15 overexpression not only induced miR-152 expression levels, but also decreased KIF2C and PKM2 expression levels in vivo (Fig. 7I, J).

## Discussion

Breast Cancer is one of leading causes of both cancer incidence and cancer death in female worldwide [1–3]. Chemotherapy is commonly used for patients who receive surgical treatment to prevent the recurrence or metastasis of breast cancer. However, chemoresistance remains to be major hurdle for many breast cancer patients, which may account for the treatment failure of breast cancer patients [2, 28]. To understand new mechanism of chemoresistance, we found that decreased expression of the transcription factor, TBX15 was associated with DOX resistance through regulating autophagy and glycolysis in cancer cells. TBX15 was initially identified as one of genes associated with genome-wide DNA methylation in several types of cancer tissues [6–8]. TBX15 expression regulated tumor development [6, 8, 11, 29]. However, the role of TBX15 on chemoresistance is still unknown. DNA hypermethylation of CpG islands may be involved in chemoresistance in certain cancer [21, 30]. Our study showed that lower expression levels of TBX15 were detected in breast cancer tissues than normal tissues using TCGA database. Thus, we want to determine whether TBX15 may affect DOX resistance in breast cancer cells. TBX15 forced expression promoted cell apoptosis and increased sensitivity to DOX treatment, while TBX15 inhibition promoted cell growth and increased resistance to DOX. In addition, TBX15 abrogated DOX resistance by inhibiting autophagy and glycolysis, a new role of TBX15 in DOX resistance.

Autophagy is known to be involved in chemoresistance of cancer cells [25, 31]. In this study, we found that TBX15 regulated autophagy which may be one of potential mechanisms of TBX15 in regulating DOX resistance. We showed that TBX15 levels were correlated with miR-152 expression levels in breast cancer tissues using bioinformatics analysis. Recently, several miRNAs were found to effectively suppress chemoresistance in cancer cells [32, 33]. Recent studies by our group showed that miR-152 overexpression sensitized cisplatin-resistant ovarian cancer cells [24]. In the present study, we observed that miR-152 overexpression mimicked the effects of TBX15 on inhibiting DOX resistance and autophagy. In addition, miR-152 inhibition made the cells sensitive to DOX treatment, the opposite effect of TBX15 in the cells. Thus, TBX15/miR-152 pathway may play an important role in DOX resistance in breast cancer.

Our recent studies have shown that miR-152 suppression induced tumor growth and angiogenesis [12, 24]. In this study, we identified that miR-152 may directly target and decrease KIF2C expression for inhibiting DOX resistance in breast cancer. KIF2C catalyzes microtubule disassembly by inducing or stabilizing the curved tubulin conformation [13]. However, the role and mechanism by which KIF2C promotes chemoresistance is unclear in breast cancer. We found that KIF2C was highly expressed in DOX-resistant breast cancer tissues and cells, and that KIF2C overexpression decreased DOX sensitivity, in part through autophagy in breast cancer cells. Higher expression levels of TBX15 and miR-152 were associated with decrease of tumor growth, lower expression of KIF2C, and more sensitivity to DOX treatment in both vivo and vitro. To further understand mechanism of KIF2C in inducing DOX resistance, we found that KIF2C directly bound to PKM2 for increasing PKM2 expression, while TBX15 overexpression abrogated this binding. Since ubiquitination is crucial to modulate protein stability [34], we deteted the ubiquitination of PKM2 by co-IP experiment assay. We found that KIF2C improved PKM2 stability by suppressing its ubiquitination. Additionally, we found that Domain-2 of KIF2C was crucial for the interaction between KIF2C and PKM2; and that Domain-2 of KIF2C promotes PKM2 stability which may regulate miR-152-induced DOX sensitivity in breast cancer cells. KIF2C may be used as a new biomarker for predicting DOX resistance in breast cancer cases. The further study would be warranted to identify potential translation significance of TBX15 / miR-152 / KIF2C / PKM2 in clinical diagnosis or new treatment option for overcoming DOX resistance in breast cancer.

## Conclusions

**Fig. 8:**
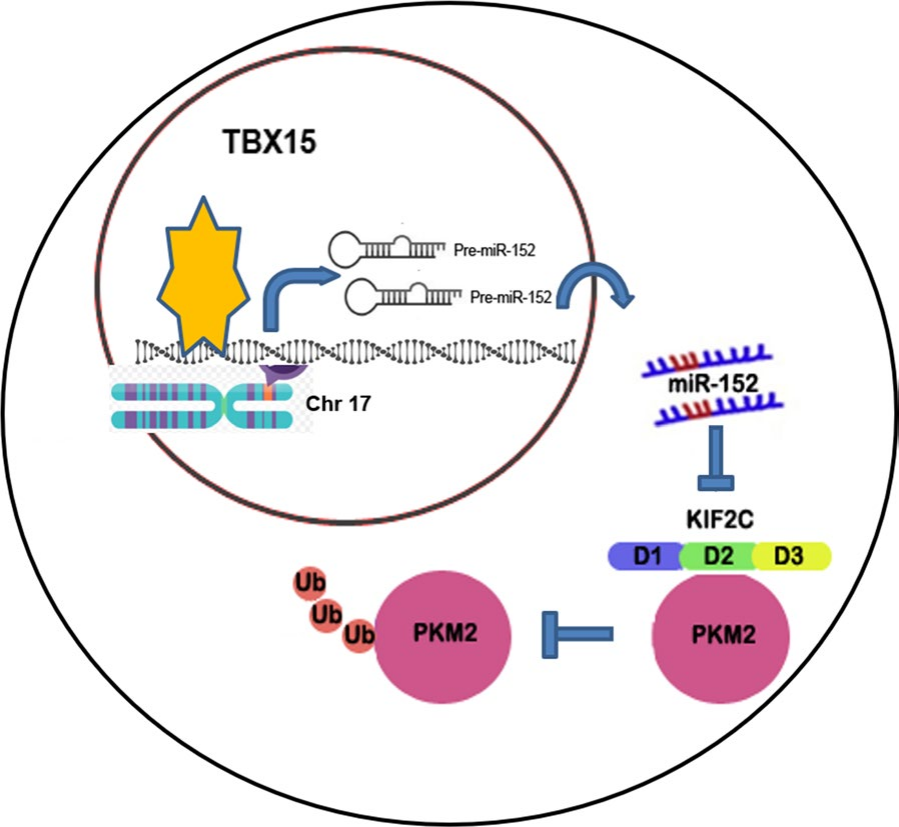
The pathway presented here was drawn out as a schematic

Our studies demonstrate that TBX15/miR-152 pathway is downregulated in DOX-resistant breast cancer tissues and that KIF2C expression is induced by miR-152 suppression. TBX15/miR-152 blocked autophagy and glycolysis in DOX-resistant breast cancer cells by targeting KIF2C. Further, KIF2C bound to PKM2 and modulated PKM2 stability to regulate autophagy and glycolysis in DOX-resistant breast cancer cells (Fig. 8). Our findings provide new insight into molecular mechanism of chemoresistance, which would be useful to develop new biomarker or therapeutic strategy for breast cancer treatment in the future.

## Supplementary Information

**Additional file 1: Figure S1.** (A) Relative expression levels of miR-152 in normal and breast tumor tissues from human patients. miR-152 expression levels were obtained from the TCGA database containing 775 breast tumors and 74 normal adjacent tissues, analyzed and presented as the means±SEM. * Indicates significant difference of the data at *P* < 0.05. (B) GSEA analysis to evaluate the correlation between expression levels of miR-152 and DOX resistance signatures using the 207 mRNA and miRNA-paired breast cancer dataset GSE22220 in the GEO database.

**Additional file 2: Figure S2.** (A) Venn diagram showing the intersection of genes identified in TCGA in breast cancer, and from potential target genes identified in the Targetscan database. Co-expression analysis between miR-152 and the intersection genes identified using the Spearman’s correlation analysis method. The red dots indicate positive correlation and the blue dots indicate the negative correlation. (B) Spearman’s correlation analysis of miR-152 and KIF2C expression breast cancer dataset GSE22220 in the GEO database. (C) Kaplan Meier plot showing predicted overall survival (OS) of breast cancer patients with high or low expression of KIF2C. (D) Bioinformatics analysis identified three domains (Domain-1, Domain-2, and Domain-3) in the human KIF2C protein (http://www.uniprot.org/uniprot/Q99661).

## Data Availability

The datasets supporting the conclusions of this article are included in this published article (and its additional files).

## Abbreviations

TBX-15: T-box transcription factor
DOX: Doxorubicin
miR-152: microRNA-152
KIF2C: Kinesin family member 2 C
PKM2: Pyruvate kinase M2
siTBX15: siRNAs targeting TBX15
siKIF2C: siRNAs targeting KIF2C
HEK-293 T: Human embryonic kidney 293 T
TCGA: The Cancer Genome Atlas
TF: Transcription factors
CHX: Cycloheximide

## References

[1] Siegel RL, Miller KD, Fuchs HE, Jemal A (2021). Cancer statistics, 2021. CA Cancer J Clin.

[2] Miller KD, Nogueira L, Mariotto AB, Rowland JH, Yabroff KR, Alfano CM (2019). Cancer treatment and survivorship statistics, 2019. CA Cancer J Clin.

[3] Bray F, Ferlay J, Soerjomataram I, Siegel RL, Torre LA, Jemal A (2018). Global cancer statistics 2018: GLOBOCAN estimates of incidence and mortality worldwide for 36 cancers in 185 countries. CA Cancer J Clin.

[4] Ni Q, Zhang F, Zhang Y, Zhu G, Wang Z, Teng Z (2018). In Situ shRNA synthesis on DNA-polylactide nanoparticles to treat multidrug resistant breast cancer. Adv Mater..

[5] Sun W, Zhao X, Wang Z, Chu Y, Mao L, Lin S (2019). Tbx15 is required for adipocyte browning induced by adrenergic signaling pathway. Mol Metab.

[6] Majumder S, Taylor WR, Yab TC, Berger CK, Dukek BA, Cao X (2019). Novel methylated DNA markers discriminate advanced neoplasia in pancreatic cysts: marker discovery, tissue validation, and cyst fluid testing. Am J Gastroenterol.

[7] Majumder S, Raimondo M, Taylor WR, Yab TC, Berger CK, Dukek BA (2020). Methylated DNA in pancreatic juice distinguishes patients with pancreatic cancer from controls. Clin Gastroenterol Hepatol.

[8] Zheng Y, Huang Q, Ding Z, Liu T, Xue C, Sang X (2018). Genome-wide DNA methylation analysis identifies candidate epigenetic markers and drivers of hepatocellular carcinoma. Brief Bioinform.

[9] Liu CT, Monda KL, Taylor KC, Lange L, Demerath EW, Palmas W (2013). Genome-wide association of body fat distribution in African ancestry populations suggests new loci. PLoS genet.

[10] Hu Z, Shi Y, Mo X, Xu J, Zhao B, Lin Y (2013). A genome-wide association study identifies two risk loci for congenital heart malformations in Han Chinese populations. Nat Genet.

[11] Gozzi G, Chelbi ST, Manni P, Alberti L, Fonda S, Saponaro S (2016). Promoter methylation and downregulated expression of the TBX15 gene in ovarian carcinoma. Oncol lett.

[12] Xu Q, Liu LZ, Yin Y, He J, Li Q, Qian X (2015). Regulatory circuit of PKM2/NF-kappaB/miR-148a/152-modulated tumor angiogenesis and cancer progression. Oncogene.

[13] Wang W, Cantos-Fernandes S, Lv Y, Kuerban H, Ahmad S, Wang C (2017). Insight into microtubule disassembly by kinesin-13s from the structure of Kif2C bound to tubulin. Nat Commun.

[14] Dai X, Hua T, Hong T (2017). Integrated diagnostic network construction reveals a 4-gene panel and 5 cancer hallmarks driving breast cancer heterogeneity. Sci Rep.

[15] Ganguly A, Yang H, Cabral F (2011). Overexpression of mitotic centromere-associated Kinesin stimulates microtubule detachment and confers resistance to paclitaxel. Mol Cancer Ther.

[16] Manning CS, Hooper S, Sahai EA (2014). Intravital imaging of SRF and Notch signalling identifies a key role for EZH2 in invasive melanoma cells. Oncogene.

[17] Zaganjor E, Weil LM, Gonzales JX, Minna JD, Cobb MH (2014). Ras transformation uncouples the kinesin-coordinated cellular nutrient response. Proc Natl Acad Sci U S A.

[18] Macintyre AN, Rathmell JC (2011). PKM2 and the tricky balance of growth and energy in cancer. Mol Cell.

[19] Israelsen WJ, Dayton TL, Davidson SM, Fiske BP, Hosios AM, Bellinger G (2013). PKM2 isoform-specific deletion reveals a differential requirement for pyruvate kinase in tumor cells. Cell.

[20] Zhou Z, Li M, Zhang L, Zhao H, Sahin O, Chen J (2018). Oncogenic kinase-induced PKM2 tyrosine 105 phosphorylation converts nononcogenic pkm2 to a tumor promoter and induces cancer stem-like cells. Cancer Res.

[21] Liu F, Ma F, Wang Y, Hao L, Zeng H, Jia C (2017). PKM2 methylation by CARM1 activates aerobic glycolysis to promote tumorigenesis. Nat Cell Biol.

[22] Tamada M, Nagano O, Tateyama S, Ohmura M, Yae T, Ishimoto T (2012). Modulation of glucose metabolism by CD44 contributes to antioxidant status and drug resistance in cancer cells. Cancer Res.

[23] Pan C, Wang X, Shi K, Zheng Y, Li J, Chen Y (2016). MiR-122 reverses the doxorubicin-resistance in hepatocellular carcinoma cells through regulating the tumor metabolism. PloS ONE.

[24] He J, Yu JJ, Xu Q, Wang L, Zheng JZ, Liu LZ (2015). Downregulation of ATG14 by EGR1-miR152 sensitizes ovarian cancer cells to cisplatin-induced apoptosis by inhibiting cyto-protective autophagy. Autophagy.

[25] Chakraborty PK, Mustafi SB, Xiong X, Dwivedi SKD, Nesin V, Saha S (2017). MICU1 drives glycolysis and chemoresistance in ovarian cancer. Nat Commun.

[26] Lee KY, Sharma R, Gase G, Ussar S, Li Y, Welch L (2017). Tbx15 defines a glycolytic subpopulation and white adipocyte heterogeneity. Diabetes.

[27] Wang X, Zhang F, Wu XR (2017). Inhibition of pyruvate kinase M2 markedly reduces chemoresistance of advanced bladder cancer to cisplatin. Sci Rep.

[28] Caetano-Pinto P, Jansen J, Assaraf YG, Masereeuw R (2017). The importance of breast cancer resistance protein to the kidneys excretory function and chemotherapeutic resistance. Drug Resist Updat.

[29] Arribas J, Cajuso T, Rodio A, Marcos R, Leonardi A, Velázquez A (2016). NF-κB mediates the expression of TBX15 in cancer cells. PLoS One.

[30] Xie VK, Li Z, Yan Y, Jia Z, Zuo X, Ju Z (2017). DNA-methyltransferase 1 induces dedifferentiation of pancreatic cancer cells through silencing of kruppel-like factor 4 expression. Clin Cancer Res.

[31] Yu T, Guo F, Yu Y, Sun T, Ma D, Han J (2017). Fusobacterium nucleatum promotes chemoresistance to colorectal cancer by modulating autophagy. Cell.

[32] Bartel DP (2018). Metazoan microRNAs. Cell.

[33] Schiewer MJ, Knudsen KE (2017). Not so fast: cultivating miRs as Kinks in the chain of the cell cycle. Cancer Cell.

[34] Enam C, Geffen Y, Ravid T, Gardner RG (2018). Protein quality control degradation in the nucleus. Annu Rev Biochem.

