# Supplementary figures and images for: TBX15/miR-152/KIF2C pathway regulates breast cancer doxorubicin resistance via promoting PKM2 ubiquitination
Source: Cancer Cell Int. 2021 Oct 18;21:542. doi: 10.1186/s12935-021-02235-w (PMC8522147; doi:10.1186/s12935-021-02235-w)

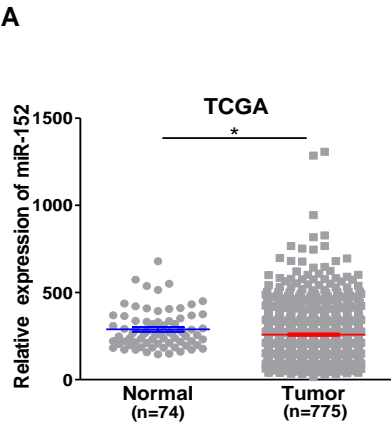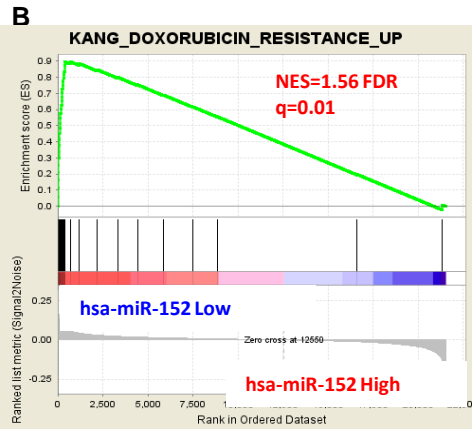

Jiang et al., Figure S1

Supplement: Supplementary file 1 — Additional file 1: Figure S1. (A) Relative expression levels of miR-152 in normal and breast tumor tissues from human patients. miR-152 expression levels were obtained from the TCGA database containing 775 breast tumors and 74 normal adjacent tissues, analyzed and presented as the means±SEM. * Indicates significant difference of the data at P < 0.05. (B) GSEA analysis to evaluate the correlation between expression levels of miR-152 and DOX resistance signatures using the 207 mRNA and miRNA-paired breast cancer dataset GSE22220 in the GEO database. [file 12935_2021_2235_MOESM1_ESM.pdf]
