# Supplementary material for: TBX15/miR-152/KIF2C pathway regulates breast cancer doxorubicin resistance via promoting PKM2 ubiquitination
Source: Cancer Cell Int. 2021 Oct 18;21:542. doi: 10.1186/s12935-021-02235-w (PMC8522147; doi:10.1186/s12935-021-02235-w)
Supplement: Supplementary file 2 — Additional file 2: Figure S2. (A) Venn diagram showing the intersection of genes identified in TCGA in breast cancer, and from potential target genes identified in the Targetscan database. Co-expression analysis between miR-152 and the intersection genes identified using the Spearman’s correlation analysis method. The red dots indicate positive correlation and the blue dots indicate the negative correlation. (B) Spearman’s correlation analysis of miR-152 and KIF2C expression breast cancer dataset GSE22220 in the GEO database. (C) Kaplan Meier plot showing predicted overall survival (OS) of breast cancer patients with high or low expression of KIF2C. (D) Bioinformatics analysis identified three domains (Domain-1, Domain-2, and Domain-3) in the human KIF2C protein (http://www.uniprot.org/uniprot/Q99661). [file 12935_2021_2235_MOESM2_ESM.pdf]

A

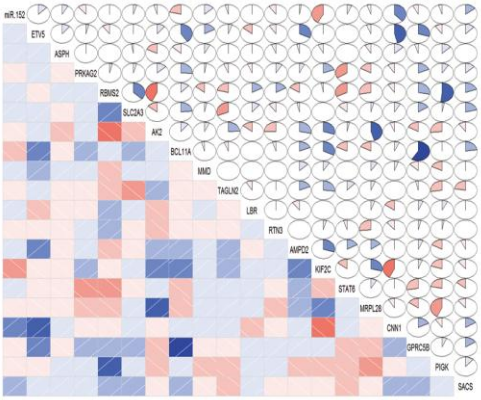

B

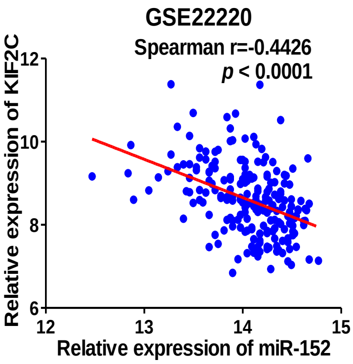

C

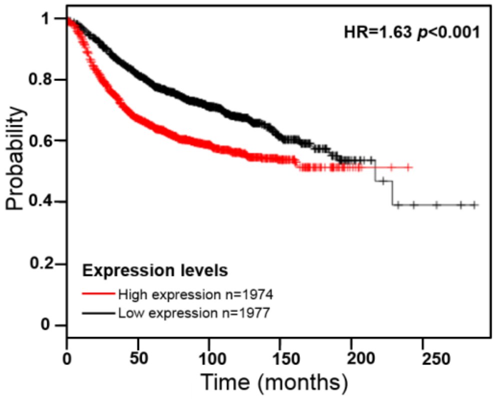

D

Domains and Repeats

Feature key	Position(s)	Description	Graphical view	Length
Region	2 – 254	Globular		253

Feature key	Position(s)	Description	Graphical view	Length
Domain	258 – 588	Kinesin motor		331

Feature key	Position(s)	Description	Graphical view	Length
Coiled coil	618 – 658			41

Jiang et al., Figure S2
